# X-box Binding Protein 1: An Adaptor in the Pathogenesis of Atherosclerosis

**DOI:** 10.14336/AD.2022.0824

**Published:** 2023-04-01

**Authors:** Tao Wang, Jia Zhou, Xiao Zhang, Yujie Wu, Kehan Jin, Yilin Wang, Ran Xu, Ge Yang, Wenjing Li, Liqun Jiao

**Affiliations:** ^1^Department of Neurosurgery, Xuanwu Hospital, Capital Medical University, Beijing, China.; ^2^China International Neuroscience Institute (China-INI), Beijing, China.; ^3^Peking Union Medical College Hospital, Peking Union Medical College, Chinese Academy of Medical Sciences, Beijing, China.; ^4^Laboratory of Computational Biology and Machine Intelligence, National Laboratory of Pattern Recognition, Institute of Automation, Chinese Academy of Sciences, Beijing, China.; ^5^School of Artificial Intelligence, University of Chinese Academy of Sciences, Beijing, China.; ^6^Department of Interventional Radiology, Xuanwu Hospital, Capital Medical University, Beijing, China.; ^7^Institute of Cerebrovascular Disease Research and Department of Neurology, Xuanwu Hospital of Capital Medical University, Beijing, China.

**Keywords:** endoplasmic reticulum stress, unfolded protein response, XBP1, IRE1α, atherosclerosis

## Abstract

Atherosclerosis (AS), the formation of fibrofatty lesions in the vessel wall, is the primary cause of heart disease and stroke and is closely associated with aging. Disrupted metabolic homeostasis is a primary feature of AS and leads to endoplasmic reticulum (ER) stress, which is an abnormal accumulation of unfolded proteins. By orchestrating signaling cascades of the unfolded protein response (UPR), ER stress functions as a double-edged sword in AS, where adaptive UPR triggers synthetic metabolic processes to restore homeostasis, whereas the maladaptive response programs the cell to the apoptotic pathway. However, little is known regarding their precise coordination. Herein, an advanced understanding of the role of UPR in the pathological process of AS is reviewed. In particular, we focused on a critical mediator of the UPR, X-box binding protein 1 (XBP1), and its important role in balancing adaptive and maladaptive responses. The XBP1 mRNA is processed from the unspliced isoform (XBP1u) to the spliced isoform of XBP1 (XBP1s). Compared with XBP1u, XBP1s predominantly functions downstream of inositol-requiring enzyme-1α (IRE1α) and transcript genes involved in protein quality control, inflammation, lipid metabolism, carbohydrate metabolism, and calcification, which are critical for the pathogenesis of AS. Thus, the IRE1α/XBP1 axis is a promising pharmaceutical candidate against AS.

## 1. Introduction

Atherosclerosis (AS) is the leading pathological cause of cardiovascular diseases and is the predominant contributor to mortality and morbidity in developed countries [1]. Increasing evidence suggests that endoplasmic reticulum (ER) stress and unfolded protein response (UPR) play critical roles in the pathogenesis of AS [2].

**Figure 1.**
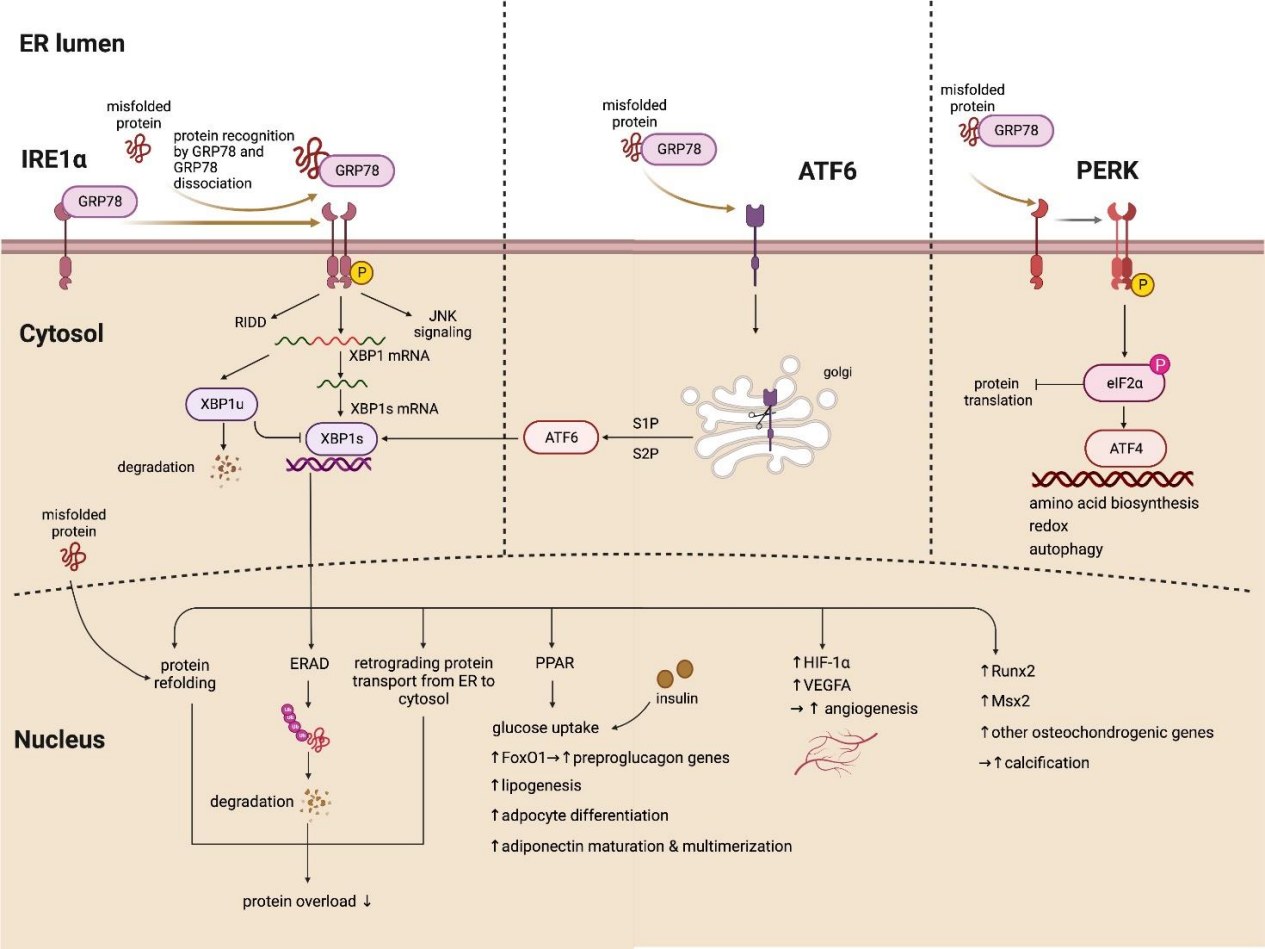
The mechanism of UPR to cope with ER stress. Three signaling cascades of UPR, inositol-requiring enzyme-1α (IRE1α), protein kinase RNA-like ER kinase (PERK) and activating transcription factor 6 (ATF6) are triggered in response to the ER stress. “→” represents promotion; “?” represents inhibition.

In the early 1990s, X-box binding protein 1 (XBP1) was first described as a mammalian ortholog of yeast hac-1, a regulator of human major histocompatibility complex (MHC) class II genes [3]. It is a transcription factor belonging to the cAMP-response element-binding/activating transcription factor (ATF) basic region leucine zipper (bZIP) family[4, 5], the gene of which is located on chromosome 22q12[6]. Currently, XBP1 is a critical regulator of UPR under ER stress [7] (Fig. 1), and is a key drug target in tumor development, especially in Myc-driven cancers [8]. Small molecule inhibitors of the inositol-requiring enzyme-1α (IRE1α)/XBP1 signaling pathway, or inhibitors of the RNase activity of IREα [9] are regarded as efficient reagents when used in combination with routine chemotherapeutic treatments [9]. Given the extensive involvement of ER stress and UPR in the pathogenesis of various diseases, alleviating ER stress is an important therapeutic strategy for diseases closely associated with inflammation and hyperlipidemia, especially AS [10]. The main purpose of this review is to elucidate the role of ER stress in atherosclerosis from the perspective of XBP1.

## 2. ER stress, UPR, and XBP1

The ER is a continuous membrane network throughout the cell that is responsible for protein modification and quality control, lipid biosynthesis, and iron storage. Any stimuli such as microenvironmental stress and abnormal proliferation disrupting the ER protein-folding capacity, leads to the accumulation of unfolded or misfolded proteins, which then induces ER stress. This perturbation of ER homeostasis can trigger adaptive mechanisms and, if the ER stress is persistent and overwhelming, a maladaptive response otherwise [11]. Molecular mechanisms, such as UPR, ER-associated degradation (ERAD), and reticulophagy, overcome ER stress at the gene expression, protein transcription, and translation levels [12].

The UPR improves protein folding and degrades misfolded proteins, mainly through three signaling cascades initiated by ER transmembrane protein sensors, including IRE1α, protein kinase RNA-like ER kinase (PERK), and activating transcription factor 6 (ATF6)[13]. Under ER non-stress conditions, these transmembrane proteins remain in an inactive state by binding to glucose-regulated protein 78 (GRP78)/immunoglobulin binding protein (BiP) in the ER lumen[14]. Under ER stress conditions, GRP78 acts as a stress sensor and initiates the UPR pathway by dissociating from IRE1α, PERK, and ATF6 [15]. Concomitantly, a direct sensing mechanism was also proposed in the IRE1α and PERK pathway, where misfolded proteins can bind to and initiate the conformational change of these sensors directly [16] (Fig. 1).

### 2.1 PERK signaling

Activated PERK transiently attenuates protein synthesis and initiates the immediate adaptation to ER stress by phosphorylating eukaryotic translation initiation factor 2 subunit-α (eIF2α) and preventing protein influx into the ER [17]. Phosphorylated eIF2α further activates the translation of a set of mRNAs involved in dephosphorylation and the restoration of protein synthesis, including activating transcription factor 4 (ATF4). ATF4 transcriptionally regulates the genes involved in redox homeostasis, amino acid metabolism, protein synthesis, and apoptosis, and also participates in a feedback loop to dephosphorylate eIF2α with the protein phosphatase 1 regulatory subunit growth arrest and DNA damage-inducible protein (GADD34) and a constitutive repressor of eIF2α phosphorylation [18]. In addition to the surveillant role of protein homeostasis, PERK triggers apoptotic cell death under chronic stress. The CCAAT/enhancer-binding protein homologous protein is regulated by ATF4 under ER stress, and promotes ER stress-induced apoptosis by modulating GADD34, death receptor 5, and the members of the B-cell lymphoma 2 or B-cell lymphoma 2-homology domain 3 only family, including NOXA [19] (Fig. 1).

### 2.2 IRE1α signaling

Inositol-requiring enzyme-1α is activated by oligomerization and autophosphorylation. Studies on the yeast homolog IRE1p show that misfolded proteins can bind to the N-terminal region directly, without GRP78 engagement. Recent *in vitro* studies have confirmed two models of yeast IRE1p activation, with GRP78 first dissociating from IRE1p, leading to its dimerization. Subsequently, the direct interaction of the unfolded protein with IRE1p can activate full ribonuclease activity [20]. In addition to the ER stress, other factors can also initiate the IRE1α pathway, including the activation of Toll-like receptors in myeloid leukocytes and the brain-derived neurotrophic factor receptors in neurons [21, 22].

Inositol-requiring enzyme-1α counteracts ER stress as an RNase and cleaves both the mRNA of the XBP1 transcription factor and a set of ER-associated mRNAs or non-coding functional RNAs to initiate the XBP1 pathway and IRE1-dependent decay (RIDD)[16]. The mechanism of the XBP1 pathway is discussed in detail later (Fig. 1). In the RIDD, mRNA abundance is downregulated mainly by the mRNA cleaving ability of IRE1α [23]. The IRE1a has several non-canonical functions that require attention. The IRE1α controls the activation of the c-Jun N-terminal kinase (JNK), extracellular regulated protein kinases (ERK), and nuclear factor kappa-B (NF-κB) pathways. The IRE1α and downstream XBP1 modulate the protein-folding load, and metabolic reaction and undergo crosstalk with other signaling pathways such as the NF-κB inflammation and mitogen-activated protein kinase (MAPK) pathways [24]. Overall, IRE1α acts by binding adaptor proteins to form a complex signaling platform at the ER membrane [20].? Moreover, a negative-feedback loop was observed between XBP1 and IRE1. The mRNA of IRE1 is significantly attenuated by spliced XBP1 (XBP1s) expression [25].

The RIDD is a conserved mechanism that maintains ER homeostasis. It targets mRNAs encoding growth-promoting proteins and the mRNA abundance is downregulated mainly by the mRNA cleaving ability of IRE1α [23]. Under ER stress, RIDD exerts the opposite effects on cell fate compared with XBP1 splicing. It decreases cell growth in a cell-specific manner. The RIDD activity increases with ER stress until apoptosis is induced [26].

There are two IRE1 paralogues in vertebrates: IRE1α and IRE1β. IRE1β is vital for mucosal homeostasis, but its influence beyond secretory cells remains unexplored [27]. It shares a relative sequence homology with IRE1α yet acts as a dominant-negative suppressor of IRE1α. Because of a nonconserved amino acid in the active site of the kinase domain, IRE1β shows attenuated XBP1 splicing activity. It can assemble with IRE1α and inhibit its XBP1 splicing activity [28]. However, IRE1β possesses preferential RIDD activity because of its stronger ability to digest 28S rRNA compared with IRE1α [28].

### 2.4 ATF6 signaling

Under ER stress, ATF6 is transmitted to the Golgi apparatus and cleaved from ATF6p90 into ATF6p50, another transcription factor belonging to the bZIP family [29]. Both XBP1s (spliced XBP1) and ATF6p50 can activate the translocation, folding, and secretion of proteins, and degrade the misfolded proteins in a concurrent manner [30]. In addition to its canonical role in protein homeostasis, ATF6 elicits protective effects against cardiac damages via non-canonical gene programming. In a recent study of a heart disease model, it was shown that ATF6 activation triggered the expression of fatty acyl-CoA reductase 1, recombinant human Ras homolog enriched in brain (Rheb) protein, and catalase, which are essential in oxidative stress regulation and growth stimuli [31, 32]. Fatty acyl-CoA reductase 1 is thought to be involved in plasmalogen synthesis, whereas the accumulation of plasmalogen is considered a negative factor in myocyte survival under oxidative stress [31]. The Rheb protein activates the growth-promoting kinase mechanistic target of rapamycin complex 1 (mTORC1). ATF6 can induce Rheb and mTORC1-dependent growth, in heart models of both the chronic exercise-induced physiological hypertrophy and pressure overload-induced pathological hypertrophy [32].

Furthermore, the overlapping downstream regulation of the IRE1α and ATF6 pathways suggests that crosstalk exists in the three UPR cascades controlled by different sensors, which enables the dynamic adjustment and coordinated expression of UPR-relevant genes under stress conditions[33] (Fig. 1).

## 3. The regulation of XBP1

### 3.1 XBP1s vs XBP1u

Mammalian cells contain two isoforms of XBP1: active and inactive. The UPR leads to the generation of active ‘spliced’ XBP1, while the inactive ‘unspliced’ form of XBP1 is dominant in unstressed cells. The conversion is mediated by IRE1α RNase activity, which cleaves the 26 nucleotides intron from unspliced XBP1 (XBP1u) mRNA and alters the open reading frame and the stop codon [34]. By the cleavage, 33 kDa XBP1u is converted to 54 kDa XBP1s, which is about 376 amino acids long, and both isoforms contain the DNA-binding domains and the nuclear localization signal (NLS). Because nuclear export-signal is cleaved, only XBP1u can shuttle in and out of the nuclear membrane [35] and serves as a dominant-negative inhibitor of XBP1s to prevent the UPR [36]. The tRNA ligase involved in XBP1 mRNA splicing, namely RtcB, can be tyrosine-phosphorylated and dephosphorylated. Tyrosine-phosphorylated RtcB failed to interact with IRE1α, further hindering XBP1 mRNA splicing. Therefore, RtcB tyrosine phosphorylation fine-tunes the IRE1α RNase regulatory network [37].

In addition to the overload of protein synthesis, an imbalance in lipid biosynthesis and the abnormal membrane fluidity can also induce the ER stress response through the IRE1α-XBP1 pathway [38]. Regarding regulation, two other pathways may also be interrelated in the modification of XBP1. For instance, ATF6 can induce XBP1 at the mRNA level [7]; however, relationship between PERK and XBP1 is still being studied [39].

### 3.2 Negative regulation of UPR by XBP1u

In addition to the transcriptional activator role of XBP1s, recent studies have demonstrated the essential role of XBP1u in negatively regulating the UPR. Here, XBP1u pre-mRNA is constitutively transcribed and translated into XBP1u, whereas XBP1u contains a degradation motif and is immediately degraded by proteasomes [40]. However, this property can be utilized by XBP1u to negatively regulate UPR, mainly by binding and degrading two transcription factors, ATF6 and XBP1s [35]. Unspliced XBP1 can recognize and associate with both XBP1s and the active form of ATF6 to form a complex that is sequestered from the nucleus, targeted by proteasomes at the XBP1u degradation domain, and further degraded into downstream transcription factors, such as p65/RelA [41]. Thus, XBP1u promptly induces negative feedback on UPR-related genes in the recovery phase after response to ER stress [42]. The switch between the activator and repressor by splicing at the mRNA level allows for quick adaptation to ER conditions [36] (Fig. 1).

### 3.3 Downstream regulation of XBP1

#### 3.3.1 Protein quality control

Spliced XBP1 plays a direct role in the UPR and mediates multiple downstream target genes, which deserves further review [43]. The UPR can either refold misfolded and unfolded proteins or activate the ERAD system to degrade them (Fig. 1). Spliced XBP1 participates in both mechanisms by specifically binding to the *cis*-acting elements, including ER stress-responsive element (ERSE) I/II and UPR elements [7]. For instance, the ER-resident protein mesencephalic astrocyte-derived neurotrophic factor (MANF) can be upregulated to reduce ER stress via XBP1s binding to an ERSE I-containing MANF promoter region [44]. In addition to the quality control of protein synthesis, XBP1s also mediates protein transportation from the ER to the cytosol to resolve protein overload [45].

#### 3.3.2 Cell survival

The UPR functions as a double-edged sword depending on its activity to counteract the elevated ER stress[46]. If the unfolded protein overload is excessive, upregulated pro-apoptotic factors, such as the C/EBP homologous protein (CHOP), outperform the activity of anti-apoptotic factors to induce cell death [47]. During sustained ER stress, XBP1s is also involved in the activation of pro-apoptotic genes. The activation of Krüppel-like factor 9 (*KLF9*) by XBP1s further promotes the expression of the inositol 1,4,5-trisphosphate receptor type 1 and the ER calcium storage regulator transmembrane protein 38 B, leading to calcium release from the ER and cell death [48] (Fig. 1). Growth arrest and DNA damage-inducible alpha 45 also induce of UPR-induced apoptosis. A potential binding site for XBP1 was identified in the GADD45A promoter [49].

#### 3.3.3 Lipid biosynthesis

Peroxisome proliferator-activated receptor α (PPARα/NR1C1), a central mediator of starvation responses, can promote fatty acid β-oxidation and ketogenesis [50]. In adipocytes, XBP1s is involved in the PPARα-mediated pathways by binding to the UPR element-like motif in the PPARα promoter. Another critical mediator of adipogenesis, PPARγ, can also be activated by XBP1s to induce insulin-stimulated glucose uptake [51]. Spliced XBP1 enhances insulin-stimulated glucose uptake by increasing PPARγ activity in adipocytes [52]. A PPARγ-activating protein, fibroblast growth factor 21 (FGF21), can be upregulated by XBP1s binding to its promoter. The FGF21-mediated activation of PPARγ by XBP1s has been demonstrated in both insulin-treated adipocytes [52] and hepatocytes [53]. The PPARγ coactivator-1α (PGC1α), an inhibitor of XBP1s expression, plays a role in hepatic gluconeogenesis [54]. It interacts with XBP1s via its activation domains, triggering the ubiquitination and degradation of the XBP1s protein. A decrease in XBP1s levels following increased PGC1α expression has been demonstrated in mouse embryo fibroblasts and mouse hepatocytes [54] (Fig. 1).

#### 3.3.3 Carbohydrate metabolism

In the livers of obese mice, the downregulation of multiple molecules is accompanied by abnormal glucose metabolism, including p38 MAPK [55] and bromodomain-containing protein 7 (BRD7) [56]. The former promotes the nuclear migration of XBP1s by phosphorylating Thr48 and Ser61. The activation of p38 MAPK reduces ER stress in severe models of obesity and diabetes in mice [55]. Bromodomain-containing protein 7 promotes the nuclear migration of XBP1s and p85α/β. In the diabetic mouse models, the upregulation of BRD7 restores glucose homeostasis and reduces blood glucose levels [56]. Dimerization of phosphotidyl inositol 3-kinase (PI3K) and p85α/β reduces the association of p85 with XBP1s, which in turn affects the nuclear migration of XBP1s. Overexpression of p85 is a common solution to this problem in obese mouse models [57]. IκB kinase beta (IKKβ) of the NF-κB pathway has also been studied in the livers of obese mice. Here, IKKβ can phosphorylate XBP1s, reduces ER stress, and improves insulin sensitivity. Nuclear factor kappa B-mediated inflammation promotes glucose homeostasis in the liver [58].

#### 3.3.4 Inflammation

Both innate and specific immunity can be modulated by ER stress [59]. Immune disorders are closely associated with ER stress, including rheumatoid arthritis, inflammatory bowel diseases, and AS [2, 60, 61]. Spliced XBP1 was first discovered and studied as a transcription factor for B cell maturation [3]. Several studies have suggested that XBP1s is involved in the differentiation and activation of various immune cells [62, 63]. In an adenovirus-mediated gene transfer model, XBP1s was transiently upregulated, which accounted for the proliferation of bone marrow-derived macrophages [64]. The duration of XBP1s overexpression determines the fate of the macrophages [64]. X-box binding protein 1 also facilitates the release of proinflammatory cytokines via NF-??B activation in macrophages [65]. The ChIP experiment revealed the recruitment of XBP1s to the promoters of inflammatory genes, including *Il6* and *Tnf* [66]. Endoplasmic reticulum (ER) stress-induced microRNAs (miRNAs) also play a bridging role in the regulation of XBP1 on immunity. The representative is miR-346, a miRNA that is significantly induced by classic stressors. Spliced XBP1 is essential for the induction of miR-346 under ER stress. The genes downstream of miR-346 are involved in immune responses, including the MHC class I gene, ER antigen peptide transporter 1, and interferon-induced genes. Thus, the activation of miR-346 is considered the cause of decreased MHC I-associated antigen presentation under ER stress [67].

## 4. UPR and XBP1 in AS

### 4.1 Atherosclerosis

**Figure 2.**
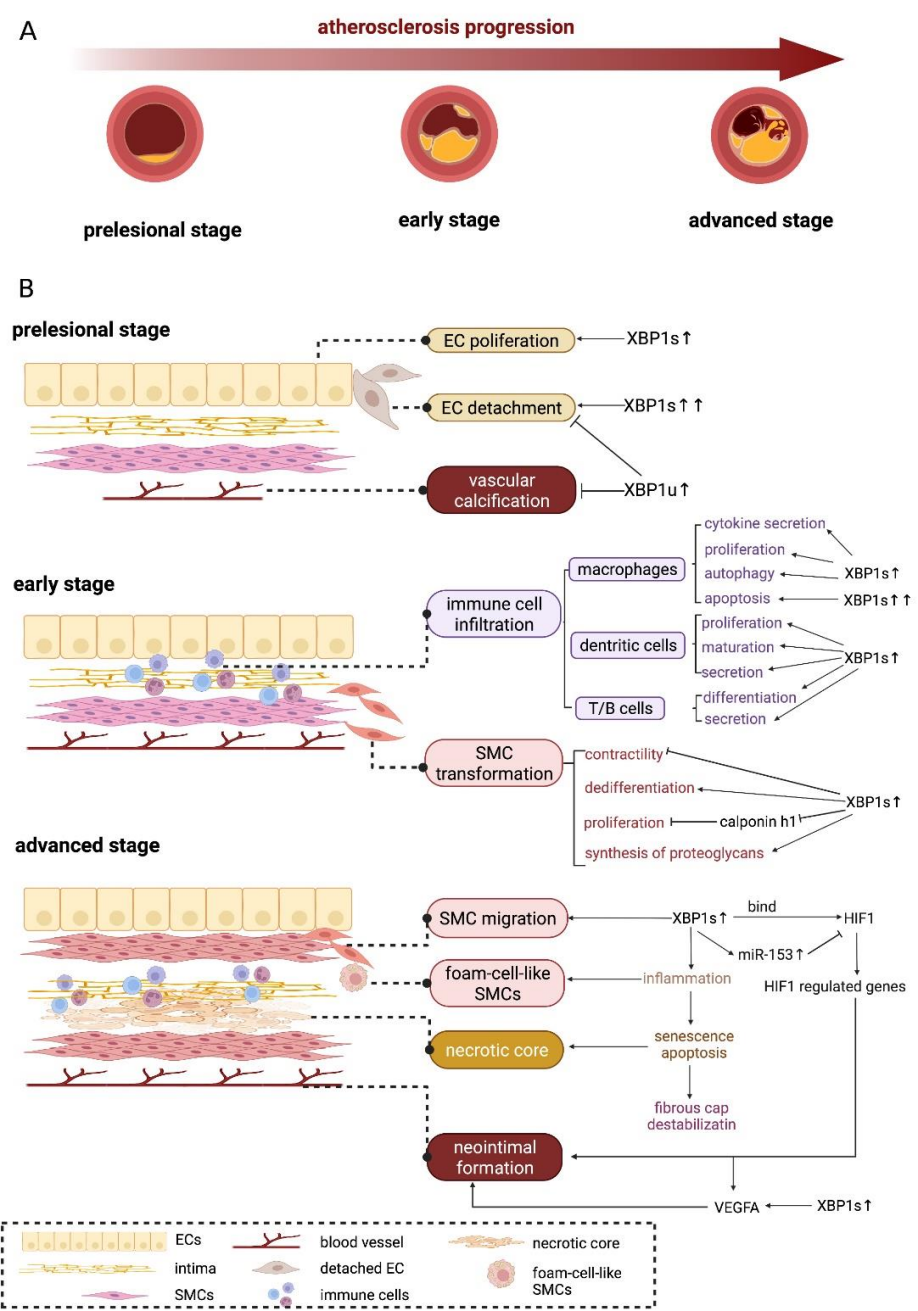
The progression of atherosclerosis and the involvement of XBP1. (A) The progression of atherogenesis can be divided into three stages, prelesional stage, early stage, and advanced stage. (B) XBP1 activation regulates the progression of atherosclerosis in a cell type-specific manner and strongly associates with the pathologic portents of atherosclerosis. “→” represents promotion; “?” represents inhibition.

Atherogenesis preferentially occurs in medium- and large-sized arteries, and its progression can be divided into three stages: prelesional, early, and advanced (Fig. 2A). Several cell types participate in the progression of the disease. In the early stages, endothelial cells (ECs) are injured due to disturbed flow, shear stress, apolipoprotein B-containing lipoproteins in the subendothelium, and other arterial wall risk factors [68]. Activated ECs attract monocytes and promote their differentiation into macrophages [2]. Macrophages internalize lipoproteins and become ‘foam cells’, triggering inflammatory responses along with other immune cells [69]. These immune cells further promote the transformation and migration of smooth muscle cells (SMCs) by producing atherogenic-stimulating signals. Activated SMCs generate a massive extracellular matrix that forms an atheroma. In early AS, the collagen-rich matrix exacerbates the disease progression by promoting the accumulation of lipoproteins and immune cells. In contrast, in advanced AS, the matrix prevents plaque rupture by forming a fibrous cap [70] (Fig. 2A). As the atheroma grows, the necrotic core is occupied by foam cells, lipids, and debris, and is covered by SMCs and the extracellular matrix. The shoulder region of the necrotic core is abundant in macrophages and immune cells, which accelerates the inflammatory response and progression of atheroma [71]. In most conditions, progression is relieved by endogenous mechanisms, including the efferocytosis of apoptotic cells by phagocytic cells, scar formation by activated SMCs, and outward remodeling of the vessel wall [72]. Defective efferocytosis leads to the formation of a necrotic core and persistent inflammation leads to plaque instability, rupture, and thrombotic vascular occlusion [73, 74] (Fig. 2B).

Endoplasmic reticulum stress and apoptosis play important roles in the pathogenesis of AS. Accumulating evidence demonstrates that the UPR is chronically activated in multiple lesional cells, including ECs, SMCs, and immune cells [2]. Pathological changes in metabolism, angiogenesis, and calcification contribute to plaque progression. Notably, persistent apoptosis leads to inflammation, vulnerable plaques, necrosis, and thrombosis [75]. The pathogenic role of ER stress in AS has been described from the perspective of different cells and pathologic portents.

### 4.2 The regulatory role of XBP1 in AS at the cellular level

Activation of XBP1 occurs throughout AS progression. In the early stage, activated by ATF6 and IRE1α, XBP1s is a representative protective molecule that mediates the ubiquitination and degradation of misfolded proteins through ERAD [76]. In addition, XBP1 mediates the upregulation of protein chaperones in ECs and upregulates the expression of genes essential for the restoration of homeostasis [76]. All these regulations improve microenvironmental stability and delay the progression of AS. However, in the advanced stage, sustained ER stress upregulates anti-survival molecules via PERK and possibly the IRE1α pathway [77]. The excessive expression of XBP1 in the late-stage triggers inflammation and necrotic core formation (Fig. 2). Thus, stage and cell type specificity could be the key to investigating the role of XBP1 in AS progression.

#### 4.2.1 Vascular endothelial cells (ECs)

Endothelial cell dysfunction is an essential contributor to AS [78]. X-box binding protein 1 is involved in the proliferation, transformation, and apoptosis of ECs via multiple downstream regulatory mechanisms[79]. The increased expression of XBP1s was observed in ECs subjected to disturbed flow *in vitro*, followed by endothelial proliferation as an adaptative response[80]. However, excessive ER stress leads to the overexpression of XBP1s, which was first demonstrated as a maladaptive reaction with endothelial detachment in cultured human veins[80]. This maladaptive reaction is achieved by coordinating XBP1s and two molecules, histone deacetylase 3 (HDAC3) and VE-cadherin [79]. Spliced XBP1 downregulates the VE-cadherin at both the transcription and translation levels. It functions as a transcriptional corepressor to the promoter of the VE-cadherin gene by indirect binding since the promoter region contains no consensus binding site for XBP1s [81]. It also degrades VE-cadherin by upregulating matrix metalloproteinases. The reduction of VE-cadherin induces caspase activation and endothelial apoptosis[80]. Histone deacetylase 3 maintains the differentiation and survival of ECs through the phosphorylation and activation of protein kinase B (PKB, also termed as Akt) [82]. It first activates phosphoinositide 3-kinases (PI3Ks), and PI3K phosphorylates Akt [80]. Moreover, XBP1u, which is typically upregulated in ECs under disturbed flow, mediates the expression of HDAC3. Knockdown of HDAC3 disrupted the regulatory effects of XBP1u under ER stress. Furthermore, there was evidence that Akt1 phosphorylation decreased after the knockdown of XBP1u, whereas the overexpression of XBP1u activated Akt1 phosphorylation. Double immunofluorescence staining and co-immunoprecipitation assays showed that the interaction between XBP1u, HDAC3, and Akt1 maintains the endothelial homeostasis under oxidative stress induced by disturbed flow [83] (Fig. 2B).

#### 4.2.2 Smooth muscle cells (SMCs)

In a wide range of disease models and physiological processes, vascular SMCs sustain the potential to reinstate gene expression patterns at the embryonic stage, namely phenotypic switching [84, 85]. Recent studies have revealed the role of phenotypic modulation in controlling plaque stability, which may be beneficial or detrimental to lesion stability. Around the pre-atherosclerotic intima, SMCs usually retain a stable phenotype with a low proliferation rate, which plays a protective role in stabilizing plaques by forming fibrous caps [86]. However, as diffuse intimal thickening develops, SMCs undergo several cellular changes, including the decreased expression of SMC markers, such as smooth muscle myosin heavy chains, as well as the reduction of contractility, lower proliferation rate, and elevated proteoglycan synthesis [87]. This alteration, termed phenotypic modulation, demonstrates the potential plasticity of SMCs in response to environmental stress, growth factors, and inflammatory mediators [85]. Prolonged phenotypic modulation leads to inflammation, the accumulation of foam cell-like SMCs, senescence, and apoptosis [88], which reduces collagen production and fibrous cap formation, consequently destabilizing the plaque [72, 89, 90]. Excessive SMC apoptosis, defective autophagy, and inefficient clearance induce a secondary necrotic core and exacerbate calcification and AS [91]. Accumulating evidence has shown that XBP1s is involved in the phenotypic alteration of SMCs. In models of both *in vivo* and *in vitro* vascular injury, XBP1s is upregulated in SMCs through the activation of the IRE1α and platelet-derived growth factor receptor β. Consequently, XBP1s promotes SMC migration by activating the PI3K/Akt pathway as well as SMC proliferation by downregulating the transcription of calponin h1. At the transcriptional level, XBP1s also suppresses the transforming growth factor (TGF)-β family [92]. Communication between SMCs and vascular progenitor cells is essential for neointimal formation; XBP1s facilitates the recruitment of stem cell antigen 1-positive (Sca1^+^)-VPC by activating type IV collagen alpha 1 (COL4A1) expression [93]. Taken together, XBP1s promotes the phenotypic modulation of SMCs and leads to neointima formation. In addition to XBP1s, XBP1u participates in vascular injury repair. An interactome analysis showed that XBP1u’s C-terminal degradation domain directly interacted with β-catenin to activate its ubiquitin-proteasomal degradation and further inhibited the Wnt signaling pathway to suppress vascular calcification [94, 95] (Fig. 2B).

#### 4.2.3 Immune cells

Immune cells, including monocytes/macrophages, T cells, B cells, and dendritic cells (DCs), play a pivotal role in AS and are regulated by XBP1 during their differentiation and proliferation. The elevation of circulating monocytes is positively correlated with atherosclerotic plaque size [96]. After infiltration, monocytes differentiate into macrophages and ingest modified lipids, predominantly modified low-density lipoprotein (LDL)[97].

Autophagy regulated by XBP1 is crucial for maintaining macrophage function. Dysfunctional autophagy leads to defective efferocytosis, cholesterol efflux, and inflammation of macrophages [98, 99], and subsequently causes the accumulation of lipid-filled macrophages, namely foam cells, and induces plaque necrosis [100]. X-box binding protein 1 regulates macrophage autophagy by transcriptionally activating autophagy-related genes [64, 65]. In an adenovirus-mediated gene transfer model, transient upregulation of XBP1s leads to the proliferation of macrophages and promotion of autophagy [64]. Another study showed XBP1 upregulated proinflammatory cytokines via NF-??B activation in macrophages [65]. Furthermore, the duration of XBP1s’ overexpression determined the fate of macrophages. Forty-eight hours of overexpression induced autophagy, whereas 72 hours triggered apoptosis; however, the precise threshold for autophagy and apoptosis needs further investigation [64] (Fig. 2B).

Dendritic cells are involved in the presentation of AS-related antigens and initiation of immune responses [101]. They also ingest modified lipids via efferocytosis or scavenger receptors and form foam cells [102]. Modified lipids, especially oxidized LDL (oxLDL), can induce the maturation and migration of DCs and antigen presentation to T cells [103], whereas excessive oxLDL can induce an anti-inflammatory response and hinder the maturation of DCs [104]. The atherosclerosis-induced alternation of DCs was mediated by XBP1s [105, 106]. X-box binding protein 1-deficient chimeric mice show plasmacytoid DCs, characterized by poorly developed ER with abnormal cisternae, as well as the downregulation of IFN-α and inflammatory cytokines. Conversely, the overexpression of XBP1s augmented inflammatory and antiviral responses in polyIC-stimulated DCs [106] (Fig. 2B).

The IRE1α/XBP1 pathway is vital for the differentiation of both cluster of differentiation (CD) 4^+^ and CD8^+^ T cells in atherosclerotic lesions[107, 108]. Here, XBP1s modulates the genes responsible for the proliferation, differentiation, cytokine production, and secretion of Th2 cells [109]. After treatment with 4μ8c, an IRE1α RNase inhibitor, a genome-wide transcriptomic analysis of Th2 cells showed that the genes associated with proliferation, cell cycle, maturation, UPR, cytokine expression, and the immune response were inhibited [109]. In the absence of TGF-β, XBP1s was shown to stimulate the production of Th17 cells and upregulate cytoplasmic calcium levels in response to environmental stress [110] (Fig. 2 B).

Different types of mature B-cells lead to different types of AS. B1 cells produce anti-oxLDL antibodies, which can be detected in both the circulation and atherosclerotic lesions of patients and are inversely correlated with the severity of AS [111]. In contrast to B1 cells, T cell-dependent B2 cells exacerbate AS via the OX40/OX40L pathway [112]. The transplantation of B2 cells into atherosclerotic mice leads to disease progression [113]. Spliced XBP1 was first proven to be indispensable for the maturation of plasma cells [114]. B cells from the *XBP1^-/-^ Rag^-/-^* chimeric mice developed normally and expressed basal levels of IgM, IgD, and B220, yet they rarely produced immunoglobulins of any isotype [115] and failed to express CD138 (syndecan-1), a marker for plasma cells [114]. Gene expression profiling indicated that XBP1s regulates genes participating in secretory pathways, including ER protein translocation across the membrane, folding, glycosylation, vesicle trafficking, and secretion[114, 116]. Notably, XBP1 acts downstream of Blimp-1, a classic transcription factor that initiates plasma cell differentiation [116](Fig. 2B).

### 4.3 The regulatory role of XBP1 in pathologic portents of AS

#### 4.3.1 Lipid metabolism

Dyslipidemia is a cause of AS. Exposure to the hyperlipidemic microenvironment leads to the accumulation of foam cells, release of inflammatory cytokines, and differentiation and infiltration of immune cells, thereby accelerating the progression of AS [117, 118].

As a cholesterol pool, the ER is sensitive to free cholesterol, and the hyperlipidemic disruption of its homeostasis can activate the UPR [119]. Among the complex signaling pathways of the UPR, the crucial role of XBP1s is not limited to maintaining ER protein homeostasis, but also fatty acid synthesis under different pathological conditions, including a high-fat diet [120], high-carbohydrate diet [121], ketogenic diet [122], fasting [122], hyperinsulinemia, and insulin resistance [121]. Studies in the hepatic lipid metabolism model have shown that the expression of XBP1s is elevated under all these pathological conditions, followed by the activation of lipogenic genes by binding to the promoter regions. A well-known regulator of the starvation response, PPARα can positively respond to XBP1s to initiate lipogenesis [121], together with acetyl-CoA carboxylase 2, diacylglycerol O-acyltransferase 2, and stearoyl-CoA desaturase 1 [120]. Additionally, XBP1 is indispensable for adipocyte differentiation. The high expression of XBP1 is a characteristic of embryonic adipose deposits [6] and white adipose cells [123], whereas the *in vitro* inhibition of XBP1 in preadipocytes causes deficient adipogenesis[124]. X-box protein 1-mediated adipocyte differentiation is initiated by CCAAT/enhancer-binding protein β (C/EBPβ) binding to the proximal promoter region of XBP1 [23], followed by the upregulation of XBP1 and activation of the pivotal adipogenic factor C/EBPα [125]. Moreover, XBP1 directs phosphatidylcholine synthesis to accelerate ER membrane expansion, which is a critical morphological response under ER stress [120].

#### 4.3.2 Carbohydrate metabolism

Glucose homeostasis is closely related to lipid metabolism and metabolic homeostasis in cells under athero-susceptible conditions. Studies in hepatocytes, pancreatic cells, and adipocytes have suggested that XBP1s is involved in glucose metabolism via the UPR- and non-UPR pathways [126].

In pancreatic α-cells, XBP1 knockdown causes insulin resistance via the phosphorylation of both IRE1α and JNK, and decreases the expression of glucagon genes via the downregulation of FOXO1, a key factor in the insulin/insulin-like growth factor 1 pathway, which can bind to the promoter of the preproglucagon genes [127]. In hepatic cells, XBP1s elevates the expression of FGF21 [53], thereby enhancing PPARγ activity to promote insulin-stimulated glucose uptake and prevent pro-inflammatory adipokine secretion [52]. In adipocytes, XBP1s directly binds to the promoters of ER chaperone genes that participate in adiponectin maturation and multimerization[128]. As an insulin-sensitizing hormone, adiponectin can promote glucose tolerance and is inversely correlated with type II diabetes mellitus [129] (Fig. 2).

#### 4.3.3 Angiogenesis

Neovascularization occurs in many physiological and pathological conditions, such as wound healing, cellular restoration in an ischemic environment, and tumorigenesis [130]. In the advanced stages of AS, angiogenesis is frequently observed in plaque formation [131] as a rescue from hypoxic and inflammatory conditions [132]. Although crucial for cellular survival in other conditions, angiogenesis is a risk factor for the advanced progression of AS because the new blood vessels are usually immature, with disorganized branching and fragile endothelial linings [133]. Abnormal vascular development and subsequential erythrocyte accumulation are also risk factors for intraplaque hemorrhaging and plaque rupture [131].

Hypoxia inducible factor (HIF) -1a is a pivotal factor in hypoxic adaptation and mediates angiogenesis by upregulating target genes in hypoxia-driven pathways [134]. Spliced XBP1 is a transcriptional cofactor for HIF1 regulated genes (*GLUT1, LOX, VEGFA, PDK1, LDHA, and DDIT4*). X-box binding protein 1 assembles the XBP1-HIF1α complex to recruit RNA polymerase II, thus activating the HIF1α mediated hypoxia response pathway genes. The XBP1 knockout has significantly downregulated HIF1α targets in breast cancer xenografts [135]. Furthermore, XBP1 induces miR-153 to degrade HIF1 as a way to fine-tune the HIF1α/vascular endothelial growth factor A (VEGFA) axis in angiogenesis. X-box binding protein 1 upregulates miR-153 by binding to the protein tyrosine phosphatase receptor type N (PTPRN) promoter, the miR-153 host gene. Therefore, miR-153 is regarded as a novel antiangiogenic therapy [136]. Inositol-requiring enzyme-1α directly (without XBP1s) stimulates HIF1 activity/expression. HIF1α is only reduced at the protein level, whereas HIF1A mRNA expression is normal. Inositol-requiring enzyme-1α-dependent decay is involved in the direct regulation of HIF1α [137].

VEGFA is another essential modulator that induces angiogenic cascades, which have been proven predominantly in tumor models [138]. In atherosclerotic lesions, VEGFA is mainly expressed by macrophages and T cells, and promotes the permeability and migration of ECs [139]. As a pivotal modulator of the IRE1α pathway, XBP1 can directly activate angiogenesis, and interact with both HIF-1a and VEGFA to activate angiogenesis as a remedy to hypoxia-induced ER stress [136, 140, 141]. Indeed, all three UPR branches (IRE1α, PERK, and ATF6) promote VEGFA mRNA expression under ER stress [142]. Spliced XBP1 activates the transcription of VEGFA through both HIF1-dependent and HIF1-independent pathways [136, 140]. Moreover, a human tumor xenograft model also showed that IRE1α/XBP1 induced a proangiogenic response in a VEGFA-independent manner [141] (Fig. 2).

#### 4.3.4 Calcification

Vascular calcification can be observed in both the intima and media, whereas arterial intimal calcification (AIC) is strongly linked to atherosclerotic lesion instability, plaque rupture [143], myocardial infarction [144], and stroke [145]. Atherosclerotic AIC is characterized by microcalcifications in the fibrous cap or deep intima, which are rich in SMCs [146]. Vascular SMCs are principally involved in calcification through the transition to osteogenic, chondrogenic, and osteoclastic phenotypes, namely calcific conversion [147]. This conversion is accompanied by the formation of calcifying vesicles, loss of SMC markers, and gain of osteochondrogenic markers, including osteopontin, osteocalcin, runt-related transcription factor 2 (Runx2), and msh homeobox 2 (Msx2) [148]. These osteochondrogenic molecules were significantly upregulated in lesions with atherosclerotic AIC, as supported by genetic lineage tracing studies in mouse models of AS [149]. In atherosclerotic lesions, infiltrated dendritic cells, macrophages, and lymphocytes express pro-inflammatory cytokines and regulatory molecules, which promote mineral deposition by triggering either apoptosis or the calcific conversion of vascular SMCs [150, 151].

The mechanism by which the UPR regulates osteogenic gene expression is conserved across cell types. The three branches of the UPR are activated to regulate osteogenic genes, both in bone development [152, 153] and atherosclerotic calcification. The expression of IRE1α, BiP, and XBP1s increased during cartilage development [152], and calcification was activated by elevated ER stress biomarkers, such as GRP78 and/or GRP94 *in vitro* [154-156]. Importantly, XBP1 directly participates in regulating transcription factors such as Osterix, which is crucial for bone formation [153]. Another study indicated that XBP1u inhibits vascular calcification in an ER stress-independent manner [95]. By interacting with the C-terminal degradation domain of β-catenin, XBP1u initiates the ubiquitin-proteasomal degradation of β-catenin, blocking the calcification axis β-catenin-Runx2/Msx2 in vascular SMCs. However, the downstream mechanisms require further investigation. Recently, autophagy has been shown to counteract vascular calcification, and the potential protective mechanisms might be related to apoptosis [157](Fig. 2).

## 5. Endoplasmic reticulum stress-targeted drugs in AS

Currently, both nonspecific alleviators of ER stress and agents specific to the UPR signaling pathways are hotspots in preclinical models. Although not always based on AS-relevant models, these reagents have the potential to treat AS [10, 158], including the analogs of endogenous molecular chaperones stabilizing protein structure [159], small molecules targeting PERK and IRE1α, and molecules promoting proteostasis via ATF6 or XBP1 signaling [160] (Table. 1).

**Table. 1:** Effects of IREα/XBP1 or ER stress modulators in experimental atherosclerosis and associated disease models.

		Disease	Model	Pharmacological effect	Ref.
Chemical chaperones					
	TUDCA	Cardiovascular diseases, especially obesity-related cardiac abnormalities	Western diet-fed Ldlr(-/-) and AMPKα2(-/-) mice model	UPR markers downregulation; global alleviation of atherosclerosis	[168]
			PDGF-induced vascular SMCs; atherogenic diet-fed rabbit model	Downregulation of ER stress mediators, including IRE1α/XBP1 pathway, BiP and Krüppel-like factor 4; global prevention of in-stent restenosis and vascular SMCs dedifferentiation	[185]
			Calreticulin-induced heart failure mice model	Inactivation of IRE1α and Xbp1 mRNA splicing; global alleviation of cardiac fibrosis	[186]
			Leptin-deficient mice model; XBP1-/- mouse embryonic fibroblasts	Insulin resistance; inhibition of obesity-induced PERK and IRE1α phosphorylation	[162]
	PBA	Cardiovascular diseases	Western diet-fed Ldlr(-/-) and AMPKα2(-/-) mice	Global inhibition of ER stress and aortic lesion development	[168]
			Western diet-fed ApoE-/- mice	Suppression of phosphorylated PERK, phosphorylated eIF2α, ATF3; alleviation of lipotoxicity	[163]
			Tunicamycin-induced THP-1 monocytes	Inhibition of ER stress, oxidative stress and apoptosis	[164]
			Leptin-deficient (ob/ob) mice model; XBP1-/- mouse embryonic fibroblasts	Insulin resistance; inhibition of obesity-induced PERK and IRE1α phosphorylation	[162]
Modulators targeting IRE1α and XBP1					
	Ginkgolide K	Cardiovascular diseases	Myocardial infarction mice model	IRE1α/XBP1 pathway activation, suppression of JNK pathway and IRE1-mediated decay; alleviation of maladaptive UPR-dependent apoptosis	[171]
	MKC8866	Cancer	Triple-negative MDA-MB-231 breast cancer cells	IRE1α RNase inhibitor, downregulation the synthesis and secretion of protumorigenic cytokines	[187]
	IRE1α RNase inhibitor 8866	Cancer	Myc-overexpressing breast tumor model	Inhibition of XBP1 acting as a synthetic lethal partner for myc	[188]
	Fulvestrant	Cancer	GH3 prolactinoma cells	Indirect IRE1α-XBP1 axis inhibition; apoptosis promotion	[189]
	Dp44mT	Cancer	Human SK-N-MC neuroepithelioma, human PANC1 pancreatic cancer and human SK-Mel-28 melanoma cell lines	Upregulation of pro-apoptotic signaling molecules; activation of IRE1α phosphorylation and XBP1 mRNA splicing	[190]
	STF-083010	Multiple myeloma	Human multiple myeloma cell lines	Inhibitor of IRE1’s RNase function; anti-multiple myeloma activity; enhancement of cytotoxicity of bortezomib	[191]
	TNFα	Airway inflammation; athsma	Tunicamycin-induced human airway SMCs	Selective activation the IRE1α/XBP1 pathway in a dose- and time-dependent fashion	[192]
	4µ8c	Under experiment phase	Mouse macrophage lines	Inhibitor of IRE1’s RNase function; inhibition of LPS-induced splicing of XBP-1 mRNA and production of IL-6 and TNF-α in macrophages	[193]
	Salicylaldehyde analogs	Under experiment phase	HEK293, MM1.s, and U266 cells; female SCID CB17 mice	IRE1 endoribonuclease inhibitors; inhibition of XBP1 splicing; downregulation of mRNAs targeted for degradation by IRE1	[173]
	Peptide derived from kinase domain of human IRE1α	Under experiment phase	Tunicamycin-induced human hepatocellular carcinoma-derived HuH7 cells and Caenorhabditis elegans experimental systems	Enhancement of IRE1α oligomerization and cleavage of XBP1 mRNA; promotion survival under ER stress	[172]
	IXA4	Under experiment phase	Diet-induced obese mice	Activator of protective IRE1/XBP1s signaling in live; improvement of systemic glucose metabolism and liver insulin action through IRE1-dependent remodeling of the hepatic transcriptome that reduces glucose production and steatosis. IXA4-stimulated IRE1 activation also enhances pancreatic function	[180]
	Quercetin	Hyperglycemia-related diseases	ox-LDL-induced RAW264.7 macrophage	Downregulation of XBP1 and CHOP; reduction of ER stress and UPR signaling in macrophages; attenuation of intracellular oxidant accumulation by inhibiting JAK2-STAT3-responsive death/survival signaling pathways; restoration of endothelial function under oxidative stress, reduction of oxLDL, adhesion molecules and inflammatory factors	[194, 195]
	Neomycin, pemetrexed, and rutin	Under experiment phase	HEK293T cell	Distortion of IRE1 RNase cavity	[178]
	Methotrexate, cefoperazone, folinic acid and fludarabine phosphate	Under experiment phase	Human cell models of glioblastoma multiforme (GBM)	Promotion of sensitivity to chemotherapy as IRE1 inhibitors	[177]
	Pumilio	Under experiment phase	Model of developing Drosophila eye	Protector of Xbp1s mRNA against RIDD	[182]

Abbreviations: AMPK, AMP-activated protein kinase Dp44mT, di-2-pyridylketone 4,4-dimethyl-3-thiosemicarbazone Ldlr, low-density lipoprotein receptor PBA, 4-phenylbutyric acid PDGF, platelet-derived growth factor TUDCA, tauroursodeoxycholic acid

### 5.1 Chemical chaperones under trials

In ER stress-related diseases, chemical chaperones are utilized to correct misfolded proteins; however, most of these are non-selective and only efficient at extremely high and even cytotoxic concentrations, which limits their clinical application [161]. Accordingly, only two chemical chaperones, 4-phenylbutyric acid (PBA) and tauroursodeoxycholic acid (TUDCA), have been tested in a mouse model of AS and have been approved for use in humans by the US Food and Drug Administration [162]. Western diet-fed ApoE^-/-^ mice treated with PBA showed significant suppression of phosphorylated PERK, phosphorylated eIF2α, ATF3, and other UPR markers, indicating the restoration of ER function in atherosclerotic lesions. In addition, PBA alleviated lipotoxicity induced by saturated fatty acids in cultured macrophages [163], as well as ER stress, oxidative stress, and apoptosis induced by tunicamycin in Tohoku Hospital Pediatrics-1 monocytes [164]. However, in an atherosclerotic model of male hamsters induced by diabetes, PBA could not alleviate ER stress or AS [165]. As a therapeutic agent against obesity-related cardiac abnormalities [166] and apoptosis in myocardial infarction [167], TUDCA also downregulated UPR markers and inhibited the progression of AS in western diet-fed AMP-activated protein kinase alpha 2 and low-density lipoprotein receptor double knockout mice [168].

### 5.2 Other promising compounds

A study using an adreno-associated virus to upregulate the expression of XBP1s demonstrated significant alleviation of ER stress in different experimental disease settings, although there are no *in vivo* tests related to AS[169]. Recent studies have developed highly selective IRE1/XBP1s activating compounds to restore ER proteostasis in the context of health and disease [170] (Table 1).

Ginkgolide, extracted from the leaves of the Ginkgo biloba tree, can activate the IRE1α/XBP1 pathway, and repress the JNK pathway and IRE1α-mediated decay to alleviate maladaptive UPR-dependent apoptosis in mouse models of myocardial infarction [171]. A specific peptide derived from the kinase domain of human IRE1α was regarded as a novel choice to promote survival under tunicamycin-induced ER stress via the enhancement of IRE1α oligomerization and cleavage of XBP1 mRNA [172]. Although unusual and challenging to apply, IRE1α RNase can also be a drug target, of which the RNase inhibitors still allow XBP1 mRNA to bind IRE1α but inhibit the catalytic cleavage in a non-competitive manner. However, there is limited information on the efficacy and specificity of these inhibitors in preclinical models [173-175].

Given the therapeutic utility of ATF6 activator in cerebrovascular diseases [176], the activators of the IRE1α/XBP1s pathway are thought to be promising in reprogramming cell proteostasis. Chevet et al. discovered novel IRE1 inhibitors among FDA-approved compounds, including cefoperazone, methotrexate, fludarabine phosphate, and folinic acid [177]. They also reported a potential way to block IRE1-mediated UPR signaling, that is, compounds that were able to bind to and distort the IRE1 RNase cavity, including pemetrexed, neomycin, rutin, and quercitrin [178]. ?The adjuvant use of IRE1 inhibitors has been reported in multiple cancers, including ?triple-negative breast cancer, ?glioblastoma multiforme, acute myeloid leukemia, and ?multiple myeloma [179].

Activators of the IRE1α/XBP1s pathway are promising candidates for reprogramming cell proteostasis. Another XBP1s-selective pharmacological IRE1 activator, IXA4, can improve protective IRE1/XBP1s signaling, systemic glucose metabolism, and liver insulin action in diet-induced obese mouse models. The IXA4-induced IRE1 activation also promotes pancreatic function. Therefore, it is considered a promising medicine for obesity-driven metabolic dysfunction with multi-tissue benefits [180]. Nevertheless, neither the inhibitor nor activator of IRE1 showed high selectivity and strong cellular activity. Recently, Ashkenazi et al. found that the allosteric activator G-1749, a compound that shares a chemical scaffold with the RNase inhibitor AMG-18, possesses high kinase-selectivity [181]. They also targeted RIDD and proposed an RNA-binding protein, Pumilio, as a protector of XBP1s mRNA against RIDD [182].

### 5.3 Prospects of drugs targeting AS

Rapid progress has been made in therapeutics for AS, but several questions remain unanswered. The anti-atherogenic mechanisms of most newly developed drugs still require elucidation. For instance, although chemical chaperones have the potential to treat AS, a clear definition of the relationship between ER stress alleviation and anti-atherogenic mechanisms has not yet been elucidated for either PBA or TUDCA [183].

Inositol-requiring enzyme-1α pharmacological approaches have been widely studied in cancer treatment. However, drugs that target AS require development. As mentioned above, compounds targeting IRE1 showed little high selectivity and strong cellular activity. Although XBP1s functions primarily as a protein homeostasis regulator, it also modulates other processes such as lipid metabolites and cell growth, and is universally expressed in all types of cells in the vessel, indicating regulatory pathway diversity and pleiotropic roles of XBP1 in AS. Current drugs that influence complex UPR networks often act on multiple pathways at the same time and cause a wide range of adverse effects, which may be solved by further identification of specific targets and organelle-specific or organ-specific therapies.

Epigenetic regulation has been a research hotspot in the past decade, but the mechanisms remain complicated in different cell types and isoforms, as well as in different stages of atheroprogression. To date, epigenetic therapies have been utilized in treating cancer, while several attractive proposals, such as combinatorial therapy and delivery of miRNA mimics, have not yet been confirmed as the experimental results are preliminary [184].

In conclusion, the exploration of anti-atherogenic mechanisms, consideration of the accuracy and efficiency of drugs, and attention to the cutting-edge development of epigenetics will shed light on the design of strategic anti-atherogenic treatments.
